# Research on Position and Torque Loading System with Velocity-Sensitive and Adaptive Robust Control

**DOI:** 10.3390/s22041329

**Published:** 2022-02-09

**Authors:** Zijia Li, Guangrong Chen, Chenyang Zhang

**Affiliations:** 1 School of Mechanical, Electronic and Control Engineering, Beijing Jiaotong University, Beijing 100044, China; 19221070@bjtu.edu.cn (Z.L.); 19221298@bjtu.edu.cn (C.Z.); 2 Robotics Research Center, Beijing Jiaotong University, Beijing 100044, China

**Keywords:** velocity-sensitive, adaptive robust, position servo, torque loading

## Abstract

In this paper, the research emphasis focuses on the tracking precision of position loading and torque loading systems with velocity-sensitive and adaptive robust control. Compared with conventional PID control, the improved PID control based on velocity-sensitive fully manifests its superiority to position loading. By contrast, we analyzed the possible influence for the control difference of conventional PID and velocity-sensitive with PID. Furthermore, for the purpose of accurate torque loading, a mathematical model was established through dynamics analysis and the adaptive robust controller, where the adaptive robust control algorithm is designed to generate the reference position trajectory for the servo system in the upper controller while closed-loop position tracking is performed in the underlying controller, was built based on a state space equation. In the end, an experimental platform was built to verify the feasibility and advantages of the position and torque loading system with the velocity-sensitive and adaptive robust control.

## 1. Introduction

Position and torque loading systems have been considered widely in recent years due to their advanced test functionality. For applications where high precise control performance is required, including flight simulator motion control [1], armament control [2], position and force servo (kinetics and dynamics) of legged robot [3,4], mobile robot system [5], manipulator control [6] and other fields, high precision controller development is receiving increasing attention in the academic world. One challenge of general control systems is that they have difficulty achieving precise position tracking and torque loading tracking due to the uncertainties and nonlinear influences of the drivers, motor body, friction and unmodeled dynamics, interference and transducer noise, etc. Therefore, the research on precise loading of position and torque is shedding further light.

For precise position loading, Chen proposed a method in [7] combining torque compensation with friction estimation that is transmitted from load side to motor side for reference and revision, using an adaptive friction observer based on modified LuGre model to effectively suppress the friction effect in the indirect drive mechanism and improve load side tracking performance. However, this method cannot determine the friction parameters of model, and the ability of compensation is limited. That is, the tracking precision is limited. Wang proposed a neural adaptive PID controller in [8] to solve the precise position tracking failure caused by nonlinearity. Intelligent control methods similar to neural network and fuzzy PID [9] can significantly reduce the static and dynamic errors of tracking, but the high computational cost also becomes the main factor limiting its use. In [10], an improved sliding mode observer (SMO) for adaptive identification based on adaptive control and improvement of cut-off frequency is proposed to fulfill high-precision positioning control of the position servo system with a variable-speed or variable-load. In [11], four realization structures of LADRC in position control of AC system are discussed, and simulation analysis is made on tracking performance. The reference [12] focuses on the influence of the PWM frequency on the dynamics of control loops and position stability and the reference [13] designs Kalman filtering methods based on the extended dynamic model using the fusion of multiple sensor signals, which may be of value to the stability of the proposed controller. In [14], Lee proposed a fuzzy s-curve profile switching method that replaces profile with a more suitable profile for precise driving of the system. Additionally, Younggi Lee proposed a compensation method for precision position-sensorless control using high-frequency pulsating voltage injection [15]. The above methods are effective for precision control but each has certain limits. Aleksej Kiselev extended generalized predictive control (GPC) algorithm [16] based on Laguerre function to track the previously known reference trajectory, which has better tracking performance than field oriented control (FOC) [17,18,19], the standard position control strategy of PMSM, but due to the influences of external interference, model parameter mismatch and finite prediction to time domain, predictive control does not work well on the global system, and the system performance is affected. In addition, the studies on the gain-scheduling control approach are provided. A multiple friction factor model is adopted in [20] using a switched linear parameter-varying (LPV) modeling approach, showing a notable improvement in the system response performance and robustness. One reference [21] proposes the use of a fuzzy gain scheduling (FGS) adaptation mechanism to tune set-point weighted PID controller for the CSTR process, which achieves positive set-point tracking and disturbance rejection. Additionally, both proposed methods are similar in terms of adaptive control to address varying parameters.

Numerous studies on precise force control are also emerging. In [22], instead of conventional strategies typically using actuated winches with torque-controlled servomotors directly connected to the cabled drum, Einar Ueland et al. have studied and demonstrated how position-controlled servomotors connected to cabled drums via clocksprings with the purpose of good force-tracking performance on moving objects, where a feedforward force controller is proposed to compensate for damping, angle-dependent force changes, delay, and non-constant clock spring characteristics. Nevertheless, the precision of force control is still limited. Weerapong Chanbua tested and compared cylinder force tracking of PID and fuzzy PD control systems under friction compensated in [23], which proves the superiority of fuzzy PID in force tracking control. Similarly, the projection algorithm in [24] and backtracking method in [25] of adaptive robust control plays a crucial role in precise loading, but the high computational cost is still a non-negligible issue. Liu presented a position-based force control scheme for tracking a moving target [26], but it does not apply to variable force tracking. In [27], Xu et al. proposed a strategy for adjusting the admittance controller parameters adaptively to solve the issue of unstable robot force control, which may be a feasible method for torque loading stability. The reference [28] gives the research on SVPWM direct torque control to improve loading precision, while a composite torque regulator is established to optimize the torque tracking performance in direct torque-controlled motor drives [29], which needs to consider variation of stator resistance, flux linkage and torque hysteresis, etc., leading to the complexity of design. Zhao et al. designed the force servo controller based on STM32 processor according to loading speed, high precision and strong stability [30]. Although the designed system has a short step response time, its tracking precision is relatively finite as the above methods.

**Figure 1:**
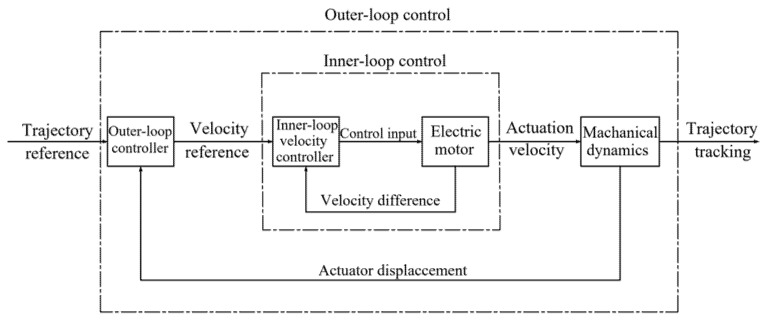
Trajectory tracking structure based on velocity sensitivity.

Thus, it cannot be denied that the simplicity of the design procedure and the low computation load have become an important criterion for the performance of precise torque and position loading system. In this paper, a method based on velocity-sensitive (Figure 1) and adaptive robust control is proposed to study the position loading and torque loading system. This paper is organized as follows. Section 2 summarizes the model of the loading system for the purpose of controller design. Section 3 introduces the controller with velocity-sensitive and adaptive robust control, including position loading control and torque loading control. Section 4 builds the position loading experimental platform, where conventional PID control and velocity-sensitive with PID are compared and the factors of performance variations are discussed, and a torque loading experimental platform consisting of determinate loading experiment and load shedding and adding experiment to verify the precision of torque loading. The main conclusions are given in Section 5.

## 2. System Model

**Figure 2:**
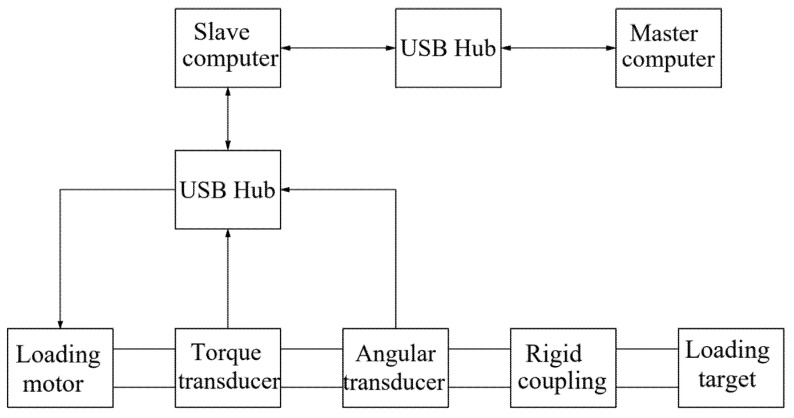
Structure of torque and position loading system.

The position and torque loading system is shown in Figure 2, including loading motor, torque transducer, angle transducer, coupling, slave computer and master computer. The working principle is that the left side of the loading motor drives the whole shaft to rotate, which leads to the rotation of the other side of the shaft. In this process, the slave computer is responsible for collecting data and communicating with the master computer (PC), the torque transducer and angle transducer reads real-time data and responds to PC by the slave computer. Meanwhile, this requires designing a program in the PC to realize the precise control of the device under the loading motor.

The dynamics of the torque and position loading system can be written as

(1) $$\begin{array}{l} M\ddot{q}+C\dot{q}+G=T_{c}-T_{e}=T^{*}\\ T_{c}=R\tau_{m} \end{array},$$

where $M\in {\mathbb{R}}^{n\times n}$ denotes the mass matrix; $C\in {\mathbb{R}}^{n\times n}$ denotes the matrix of Coriolis and centrifugal terms; $G\in {\mathbb{R}}^{n}$ denotes the gravity terms; $T^{*},T_{c},T_{e}\in {\mathbb{R}}^{n}$ donate the net, control and environmental force/moment vector of rigid body, respectively; R is the normalized quantity of the transmission ratio parameters of the reducer and the dimension parameters of the measuring device, and its value is an uncertain quantity, which needs to be confirmed according to the installation site of the device; and $\tau_{m}$ is the output torque of motor. Note that $T_{e}$ is the usual loading torque.

Considering that the precision of the motor with axial torsional dynamics will be affected by loads torque and other factors during operation [31], as well as the existence of sensor zero bias, noise and other unexpected factors that affect the precision, the following loading model is established [32]:

(2) $$T_{me}=T_{c}-T_{e}=k\left(\frac{q_{m}}{R}-q_{e}\right)+\tilde{D}=k\left(\frac{q_{m}}{R}-q\right)+\tilde{D},$$

where $q_{m}$ is the rotation angle of motor, $q=q_{e}$ is the rotation angle of loading system, $\tilde{D}$ is the term of integrated unmodeled dynamics and external disturbances, and $T_{me}$ is the difference between the control output torque of reducer and loading torque. When $T_{me}=0$*,* the desired loading torque is tracked well. $q_{m}$ and q are the coupling positions at both ends, and the two ends of the coupling are the output end of the reducer and the shaft of the measured device.

Considering the time delay change and the linear change of load-magnitude by the torque transducer, the torque integral is used as a quantity of error analysis for control design, and the following model can be obtained:

(3) $$\frac{d}{dt}\int_{0}^{t}T_{me}dt=k\left(\frac{q_{m}}{R}-q\right)+\tilde{D}.$$

In order to obtain uncertain parameter values under different installations and reducer transmission ratios using an adaptive algorithm, normalized interference and normalized unknown parameters are defined as follows:

(4) $${\tilde{\Delta }}_{1}=\frac{k}{R}q_{m}+{\tilde{D}}_{1},$$

(5) $$\theta_{1}=\frac{k}{R},$$

(6) $$\theta_{2}=\Delta_{1n},$$

(7) $${\tilde{\Delta }}_{1}=\Delta_{1}+\Delta_{1n},$$

where $\Delta_{1n}$ is the constant part of ${\tilde{\Delta }}_{1}$, and $\Delta_{1}$ is the time-varying part of ${\tilde{\Delta }}_{1}$.

In order to facilitate the analysis, the following hypothesis is made and verified [32]:

The range of unknown parameter θ and uncertain nonlinear term ${\tilde{\Delta }}_{1}$ is determined. Define $\theta ={\left[\begin{array}{cc} \theta_{1} & \theta_{2} \end{array}\right]}^{T}$,

(8) $$\theta \in \Omega_{\theta }≜\left\{\theta \right.:\theta_{\min}\leq \theta \leq \left.\theta_{\max}\right\},$$

(9) $$\left|{\tilde{\Delta }}_{1}\right|\leq \delta \left(t,x\right),$$

where $\theta_{\min}={\left[\theta_{1\min},\theta_{2\min}\right]}^{T}$, $\theta_{\max}={\left[\theta_{1\max},\theta_{2\max}\right]}^{T}$, $\delta \left(t,x\right)$ are known.

Then, defining the following state variables:

(10) $$x_{1}=\int_{0}^{t}T_{me}dt,$$

(11) $$x_{2}=q,$$

(12) $$x={\left[\begin{array}{cc} x_{1} & x_{2} \end{array}\right]}^{T}.$$

Based on the above deduction, the state space equations are established as follows:

(13) $${\dot{x}}_{1}=-\theta_{1}x_{2}+\theta_{2}+\Delta_{1},$$

(14) $$y=x_{2}=u.$$

## 3. Improved PID Control Based on Velocity-sensitive for Position Loading

### 3.1. Conventional PID Controller

The PID control algorithm consists of proportion (P), integral (I) and differential (D). PID controller (PID regulator), an automatic controller, is widely used on account of the advantages of simple principle, easy realization, wide application, independent control parameters and simple parameter selection. Moreover, it can be proved theoretically that PID controller is an optimal control for the typical control objects of “first-order lag + pure lag” and “second-order lag + pure lag”. PID regulation law is effective for dynamic quality correction of a continuous system, whose parameter setting mode is simple and structure change is flexible (PI, PD, etc.).

**Figure 3:**
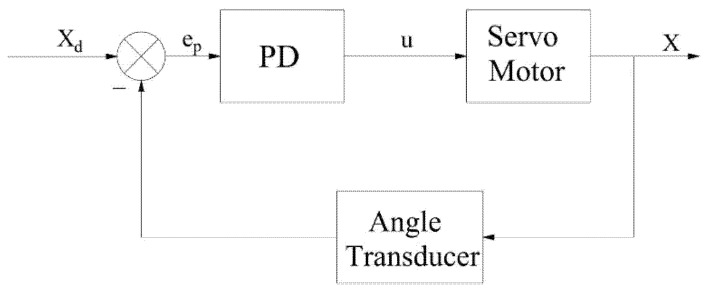
Trajectory tracking with traditional PD.

**Figure 4:**
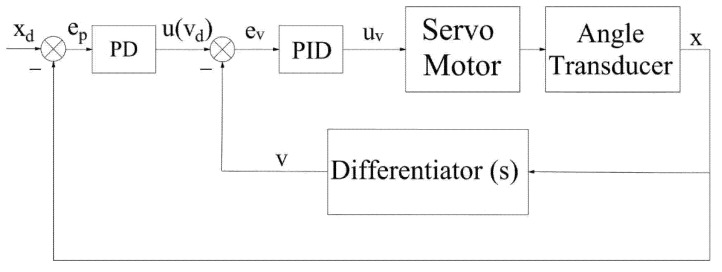
Trajectory tracking with velocity-sensitive PID.

Position loading with traditional PD control is shown in Figure 3. The angle transducer, it should be noted, is used to measure the loading motor shaft rotation angle so that the actual position x can be obtained. Moreover, the angle transducer in Figure 4, in addition to playing the same role as above, can also indirectly provide the rotation velocity of the motor output shaft, or rather, the angle measured by angle transducer can be transformed into velocity in the upper computer.

In the system of Figure 3, traditional PD control eliminates the error by controlling the error between the target position $x_{d}$ and the actual position x of the loading motor. The specific formula is as follows:

$$e_{p}=x_{d}-x,$$

(15) $$u=K_{p1}\cdot e_{p}+K_{d1}\cdot {\dot{e}}_{p},$$

where $e_{p}$ is the error of position, u is the input, $K_{p1}$ is proportional regulation coefficient, and $K_{d1}$ is differential regulation coefficient.

### 3.2. Velocity-Sensitive with PID

The velocity-sensitive with PID control is introduced in the system of Figure 4. The velocity reference value as the input of inner loop velocity control system is generated on the basis of the trajectory reference in an outer loop system of PID control to complete the precise output of actual velocity, that is, PID algorithm to change velocity value constantly in the inner loop ends up making the actual velocity to a desired velocity requirement, which contributes to the actual displacement controlled by the outer loop system basically being consistent with the expected displacement. Finally, the precise trajectory tracking is achieved under the joint action of the double close loop control systems.

Different from the traditional PID control, the input u in (15) is taken as the expected target $v_{d}$ of the inner loop velocity PID control, which eliminates the error by controlling the error between the target velocity $v_{d}$ and the actual velocity v of the loading motor. The specific formulas are as follows:

$$v_{d}=u=K_{p}e_{p}+K_{d}{\dot{e}}_{p}+{\dot{x}}_{d},$$

$$e_{v}=v_{d}-v,$$

(16) $$u_{v}=K_{p2}e_{v}+K_{d2}{\dot{e}}_{v}+K_{i}\int e_{v}dt,$$

where $e_{v}$ is the error of velocity, $u_{v}$ is the input, $K_{p2}$ is proportional regulation coefficient, $K_{d2}$ is differential regulation coefficient, and $K_{i}$ is integral regulation coefficient.

### 3.3. The Stability of Velocity-Sensitive Controller with PID

The system of interest in (13) and (14) is a general nth order nonlinear control inputs affine system [33], which can be given by the following equations

(17) $$\dot{x}=f(x)+G(x)u+d,$$

y=h(x),

where $f\in {\mathbb{R}}^{n}$ is a vector field. $G\in {\mathbb{R}}^{n\times m}$ is the effectiveness matrix of control, u is the control input, d is a continuous external disturbance, y is the output. x, d, and h are assumed continuous, f and G are assumed to be $C^{\infty }$ functions of x and all degrees of differentiation are bounded.

Let h(x)=x, and the system relative degree is ${\left(1,\ldots ,1\right)}_{1\times n}$, then the first-order time derivative of the output is

(18) $$\dot{y}=\dot{x}=f(x)+G(x)u+d.$$

The system dynamics in (18) can be rewritten by applying the Taylor series expansion at the beginning instant of each sampling interval (denoted by subscript 0).

(19) $$\dot{x}={\dot{x}}_{0}+G(x_{0})(u-u_{0})+\frac{\partial [f(x)+G(x)u]}{\partial x}\left|{}_{0}(x-x_{0})\right.+(d-d_{0})+O({\left(x-x_{0}\right)}^{2}).$$

The first two terms are an incremental nominal part, and the last three terms are a perturbation part. Setting the last three terms of (19) as

(20) $$\delta (\Delta x,\Delta d)=\frac{\partial [f(x)+G(x)u]}{\partial x}\left|{}_{0}(x-x0)\right.+(d-d0)+O({\left(x-x0\right)}^{2}).$$

Then, (20) is written as

(21) $$\dot{x}={\dot{x}}_{0}+G(x_{0})(u-u_{0})+\delta (\Delta x,\Delta d).$$

Using the continuity of x and d and the boundedness of the differentiation of G(x), the limits of (21) as the time increment T goes to 0 is calculated as

(22) $$\underset{T\to 0}{\lim}\delta (\Delta x,\Delta d)=0.$$

With a fast sampling rate, the δ approaches zero. Thus, in every sampling interval, the control law is

(23) $$u=u_{0}+G^{-1}(x_{0})(\upsilon -{\dot{x}}_{0}),$$

where ${\dot{x}}_{0}$ means the rotation velocity at the beginning of every sampling interval, instead of a fixed reference point, and it is obtained by angular transducer measurement and updated in every sampling period. v is defined as a continuous change as Figure 4.

Substituting (23) into (21), the closed-loop system dynamics are given by

(24) $$\dot{x}=\upsilon +\delta (\Delta x,\Delta d).$$

Combining (22) and (24), it is clear that the system is linearized under an infinitesimal sampling time. Based on (16) and Choosing $\upsilon =v_{d}=K_{p}(x_{d}-x)+K_{d}({\dot{x}}_{d}-\dot{x})+{\dot{x}}_{d}$, the dynamics of system error can be written as

(25) $${\dot{e}}_{p}=-{\left[K_{d}+I\right]}^{-1}K_{p}e_{p}+{\left[K_{d}+I\right]}^{-1}\delta (\Delta x,\Delta d),$$

where $-{\left[K_{d}+I\right]}^{-1}K_{p}$ is Hurwitz. The origin of (25) is globally exponentially stable with an infinitesimal T in (22), and it can be also proved by Lyapunov stability theorem.

However, the small sample time T is a finite value in real applications. It can be found that $\forall \varepsilon >0,\exists T>0,s.t.{\left\|\delta (\Delta x,\Delta d)\right\|}_{2}\leq \varepsilon$ in (22). Then, its stability analysis can be given by Lemma 1 [33], and the proof of Lemma 1 has been given in Lemma 13.4 in [34].

**Lemma** **1.** **[33].**  *Consider the closed-loop system in (25), where* $-{\left[K_{d}+I\right]}^{-1}K_{p}$*is Hurwitz, if*${\left\|\delta (\Delta x,\Delta d)\right\|}_{2}\leq \varepsilon$*, the error*e*will be globally ultimately bounded by*εc*for some*c>0.

## 4. Adaptive Robust Controller for Torque Loading

**Figure 5:**
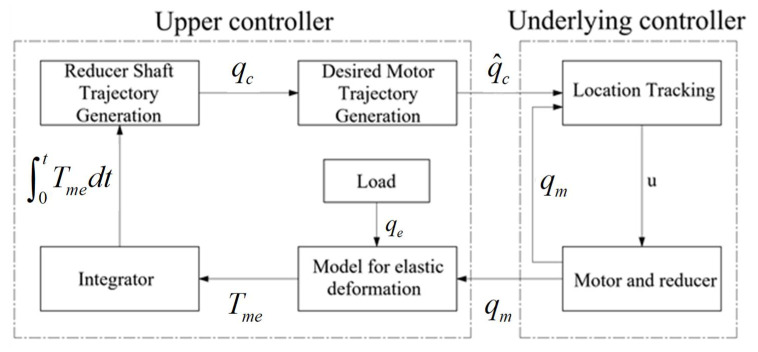
Model of adaptive robust controller.

**Figure 6:**
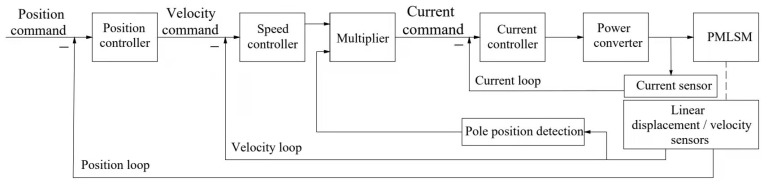
Three-loop control of underlying controller.

The adaptive robust controller is presented mainly on the premise of (12). The design of the controller contains all unknown elements and uncertainties, and its control structure is divided into two layers, the upper controller and the underlying controller, as shown in Figure 5. The upper controller receives the loading torque measured by the torque transducer and generates the position reference of the servo motor. Additionally, the underlying controller is in control of the servo motor for precise closed-loop position tracking considering the factors such as transmission ratio, which mainly includes the position, velocity and current of the motor three-loop PI controller, as shown in Figure 6.

### 4.1. Upper Controller

Due to the need of producing different deceleration ratio requirements according to the installation site of the device under test, stiffness of the output shaft and load requirements, it is necessary to estimate the uncertain parameters above online in the upper controller and resist the influence of unmodeled dynamics such as friction. In order to improve the stability of the control system, it is required to eliminate the unpredictable disturbances and unmodeled dynamics above. Then, adaptive robust control (ARC) algorithm is proposed in the design of upper controller to overcome these problems.

In (13), the main state space equation is taken as the input of system, then the control problem is transformed into designing the control law of $q_{c}$ for the output shaft to reducer position $x_{2}=q_{e}$ so that the tracking error $e_{1}=x_{1}-x_{1d}$ of torque integral is convergent or bounded. Here, $x_{1}$ and $x_{1d}$ are, respectively, the actual and desired torque integral value of the load torque tracking error to be controlled, and considering the linear loading requirements, it should be a quadratic function. $x_{1d}=0$ means the desired loading torque is tracked well, including the case when the desired loading torque is not constant.

The control law and the estimation rate of uncertain parameters are determined as follows:

$$q_{c}=q_{ca}+q_{cs},$$

$$q_{ca}=-\left(1/{\hat{\theta }}_{1}\right)\left({\dot{x}}_{1d}-{\hat{\theta }}_{2}\right),$$

$$q_{cs}=-\frac{1}{\theta_{1\min}}\left(-k_{1}e_{1}\right)+q_{csn},$$

$$\theta ={\left[\begin{array}{cc} \theta_{1} & \theta_{2} \end{array}\right]}^{T},\phi_{1}={\left[\begin{array}{cc} -q_{ca} & 1 \end{array}\right]}^{T},$$

$$\dot{\hat{\theta }}=Proj\left(\Gamma_{1}\phi_{1}e_{1}\right),$$

(26) $$Proj_{{\hat{\theta }}_{i}}({•}_{i})=\left\{\begin{array}{cc} 0 & if{\hat{\theta }}_{i}=\theta_{i\max}and{•}_{i}>0\\ 0 & if{\hat{\theta }}_{i}=\theta_{i\min}and{•}_{i}<0\\ {•}_{i} & otherwise \end{array}\right.,$$

where $q_{ca}$ is the model compensation term. $q_{cs}$ is the robust feedback term. $k_{1}>0$ is the linear feedback gain. $\Gamma_{1}>0$ is a positive semi-definite matrix with constant coefficients. $\hat{\theta }$ is the estimated value of θ. $q_{csn}$ is a nonlinear feedback term satisfying the following conditions:

(27) $$e_{1}\left(-\phi_{1}^{T}\tilde{\theta }+\Delta_{1}-\theta_{1}q_{csn}\right)\leq \varepsilon_{1},$$

(28) $$-\theta_{1}e_{1}q_{csn}\leq 0,$$

where $\varepsilon_{1}>0$ is an arbitrarily small number, whose selection affects the final convergent error of the controller. $\tilde{\theta }=\hat{\theta }-\theta$ is the parameter estimation error. Define $e_{2}=x_{2}-q_{c}$ as the position tracking error.

Then, the first error subsystem is as follows:

(29) $${\dot{e}}_{1}=-\frac{\theta_{1}}{\theta_{1\min}}k_{1}e_{1}-\theta_{1}e_{2}-\phi_{1}^{T}\tilde{\theta }+\Delta_{1}-\theta_{1}q_{csn}.$$

It is clearly seen that the interaction torque can be minimized when $x_{2}=q_{c}$. Thus, the virtual control law $q_{c}$ is regarded as the load intent inferred based on the model for elastic deformation.

The desired rotation angle qc has been obtained from the upper force controller. In the later underlying low-level motion tracking controller, the desired rotation angle and its derivatives will be used to compute partial derivatives w.r.t. time in the control law. Here, an output differential observer, given as follows, is used to estimate the desired velocity, acceleration, and jerk for the motor rotation angle [32]:

(30) $$\begin{array}{l} {\dot{\zeta }}_{1}={\dot{\zeta }}_{2}+\alpha_{1}\left(q_{c}-\zeta_{1}\right)\\ {\dot{\zeta }}_{2}={\dot{\zeta }}_{3}+\alpha_{2}\left(q_{c}-\zeta_{1}\right)\\ {\dot{\zeta }}_{3}=\alpha_{3}\left(q_{c}-\zeta_{1}\right) \end{array}$$

where ${\hat{q}}_{c}=\zeta_{1},{\dot{\hat{q}}}_{c}=\zeta_{2},{\ddot{\hat{q}}}_{c}=\zeta_{3}$ represent the estimated desired rotation angle, velocity, and acceleration, respectively. The transfer function of (30) from input qc to $\zeta_{1}$ is

(31) $$H(s)=\frac{\zeta_{1}(s)}{q_{c}(s)}=\frac{\alpha_{1}s^{2}+\alpha_{2}s^{1}+\alpha_{3}}{s^{3}+\alpha_{1}s^{2}+\alpha_{2}s^{1}+\alpha_{3}},$$

where $\alpha_{1},\alpha_{2},\alpha_{3}$ can be chosen via pole placement based on the desired closed-loop bandwidth. Additionally, the three initials of the filter can be set to reduce transient errors in the low-level loop. The estimated errors of the desired rotation angle and its derivatives can be lumped into uncertain nonlinearities, which can be attenuated by robust control.

### 4.2. Underlying Controller

The underlying controller is mainly responsible for the position tracking control of the servo motor, which involves the three-loop control principle of the motor control:

The first is the current loop, which is completely carried out inside the servo driver. The output current of each phase to the motor is detected by sample resistance, then the negative feedback current is adjusted by PI of the controller, so as to achieve the output current as close as possible to the set current to control the torque of the motor.

The second is the velocity loop. The negative feedback PI is adjusted through the signal of the servo motor encoder. The output of PI in the loop is directly the setting of the current loop, so the velocity loop control includes itself and the current loop. The current loop is the root of control, in the velocity and position control at the same time the system is actually also in the current (torque) control to achieve the corresponding control of velocity and position.

The third is the position loop, which is the outermost loop and constructed between the external controller and the motor encoder or final load. The position loop receives the reference track of the upper controller and generates the setting of the velocity loop. In the mode of position control, all the operations of the three loops are performed.

### 4.3. The Stability of Adaptive Robust Controller

**Theorem** **1.**  *In the upper controller, if the zero tracking error* $e_{2}=0$*is achieved in the underlying controller (it is stability-guaranteed with PID controller), the following holds using the control law (26).*

(a) Tracking error of loading torque is bounded by

(32) $$V_{s}(t)\leq \exp(-\lambda t)V_{s}(0)+\frac{\varepsilon_{1}}{\lambda }\left[1-\exp(-\lambda t)\right],$$

where $V_{s}=\frac{1}{2}e_{1}^{2},\lambda =2\frac{\theta_{1}}{\theta_{1\min}}k_{1}$.

(b) If $\Delta_{1}=0$ after a finite time, loading torque tracking error is bounded with integral asymptotically converging to zero, i.e., $e_{1}\to 0$ as t→∞.

**Proof** **of** **Theorem** **1.** Differentiate $V_{s}$ by using (29), it yields

(33) $$\begin{array}{l} {\dot{V}}_{s}=e_{1}{\dot{e}}_{1}\\ =-\frac{\theta_{1}}{\theta_{1\min}}k_{1}e_{1}^{2}-\theta_{1}e_{1}e_{2}+e_{1}\left(-\phi_{1}^{T}\tilde{\theta }+\Delta_{1}-\theta_{1}q_{csn}\right) \end{array}$$

If $e_{2}=0$ in the underlying controller, substituting (27) and (28) into (33) yields

(34) $${\dot{V}}_{s}\leq -\frac{\theta_{1}}{\theta_{1\min}}k_{1}e_{1}^{2}+\varepsilon_{1},$$

which proves (a) of Theorem 1.

When $\Delta_{1}=0$, choose a Lyapunov function $V_{a}=V_{s}+\frac{1}{2}{\tilde{\theta }}^{T}\Gamma_{1}^{-1}\tilde{\theta }$. Differentiating $V_{a}$ yields

(35) $$\begin{array}{l} {\dot{V}}_{a}={\dot{V}}_{s}+{\tilde{\theta }}^{T}\Gamma_{1}^{-1}\dot{\tilde{\theta }}\\ =-\frac{\theta_{1}}{\theta_{1\min}}k_{1}e_{1}^{2}-e_{1}\left(-\theta 1qcsn\right)+{\tilde{\theta }}^{T}\Gamma_{1}^{-1}\left(\mathrm{Pr}oj\left[\Gamma_{1}\phi_{1}e_{1}\right]-\Gamma_{1}\left(\phi_{1}e_{1}\right)\right)\\ \leq -\frac{\theta_{1}}{\theta_{1\min}}k_{1}e_{1}^{2} \end{array}$$

Therefore, $e_{1}\in L_{2}$. It is also easy to check that ${\dot{e}}_{1}$ is bounded. Therefore, by Barbalat’s lemma, ${\dot{e}}_{1}\to 0$ as t→∞, which proves to (b) of Theorem 1. □

## 5. Experiment Validations

### 5.1. Hardware Setup

**Figure 7:**
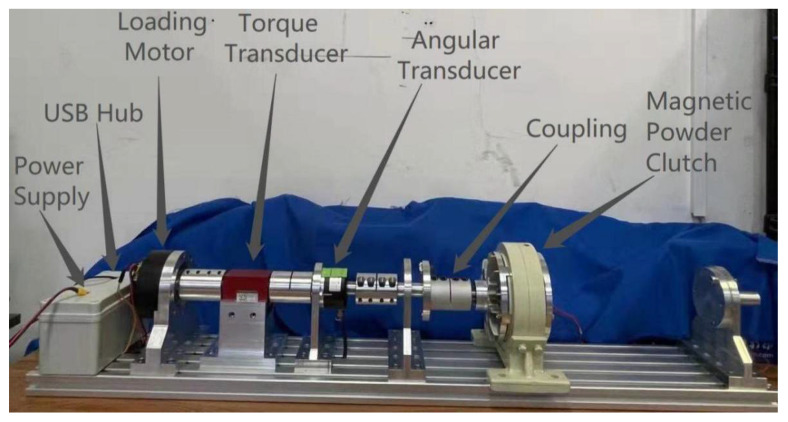
The overall structure of position and torque testing device.

The overall structure of the experimental platform is shown in Figure 7 where the magnetic powder clutch plays the role of load in the torque loading experiment, and the hardware configurations are shown in Table 1:

### 5.2. Position Control Experiment

Test method for displacement is that the loading side is not connected to other devices such as magnetic powder clutch, then entering the target position in the master computer to start the operation.

The traditional PID control and the velocity-sensitive PID control are, respectively, used for trajectory tracking, and setting the desired displacements to 100°, 200°, and 300°.

For the parameters tuning of the proposed controller, we basically adjust PID parameters by simulink of MATLAB. In this process, there are some methods involved in tuning PID gains. We used the method in [35] to determine the stability parameter set, where PID control parameters (Kp, Kd, Ki) can make the closed-loop feedback control systems stable. Furthermore, on the basis of the above stability parameter set, we also tuned the PID controller parameters under the condition of ensuring the robustness of the controller [36]. The optimal parameters were designed as follows: $K_{p1}=0.2$, $K_{d1}=0.4$, $K_{p1}=0.34$, $K_{d2}=0.33$, and $K_{i}=0.05$.

The above results of parameter tuning and the data analysis in Section 5 of this paper have both proved that the stability of the improved PID control based on velocity sensitivity.

**Figure 8:**
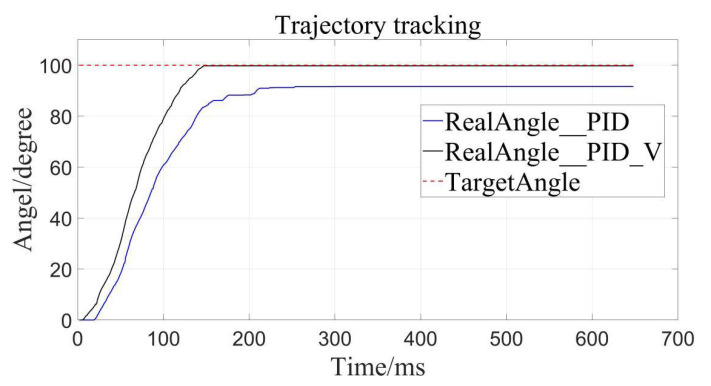
Target angle to 100°.

**Figure 9:**
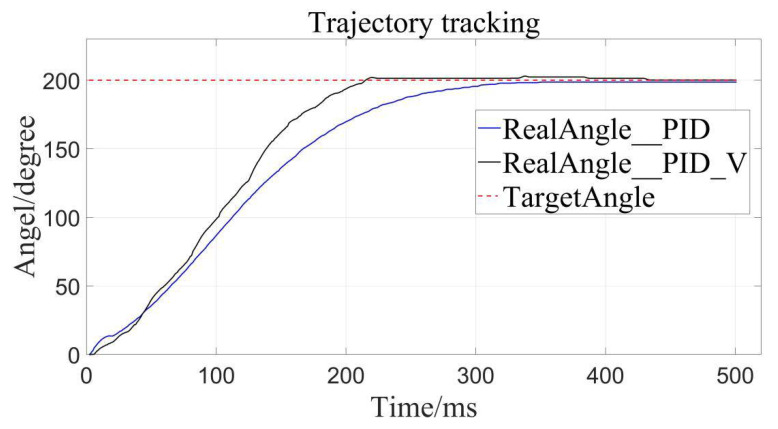
Target angle to 200°.

**Figure 10:**
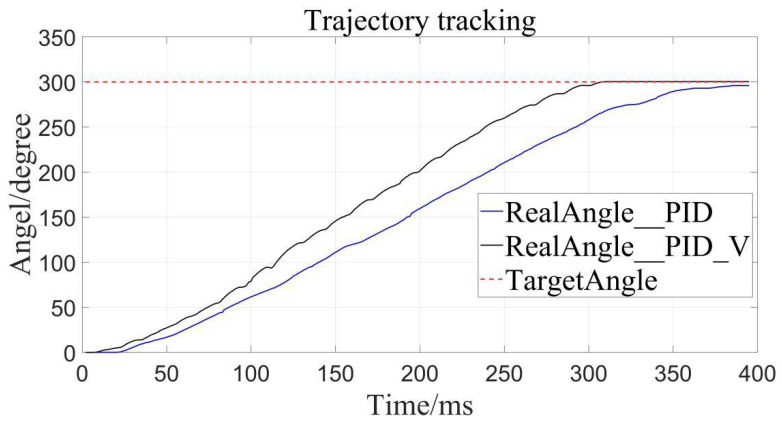
Target angle to 300°.

**Figure 11:**
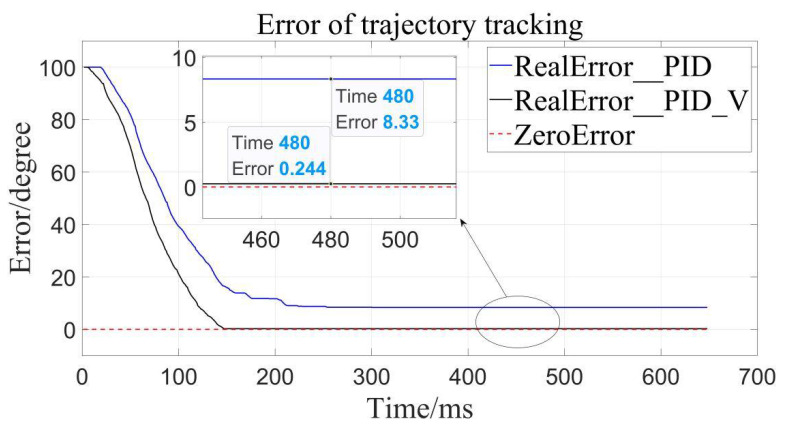
Error to target angle to 100°.

**Figure 12:**
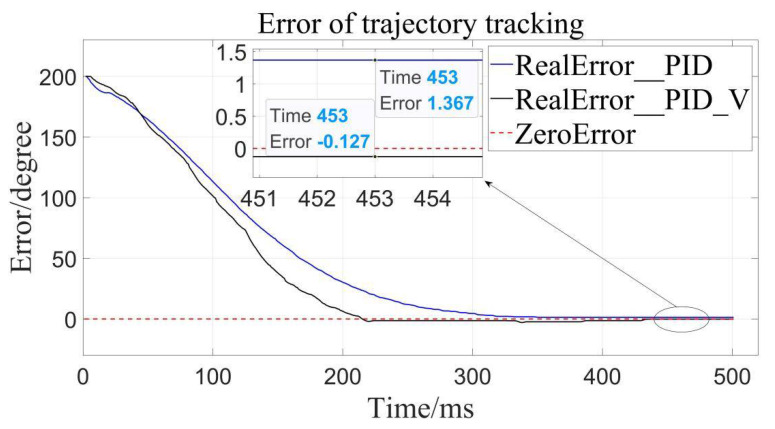
Error to target angle to 200°.

**Figure 13:**
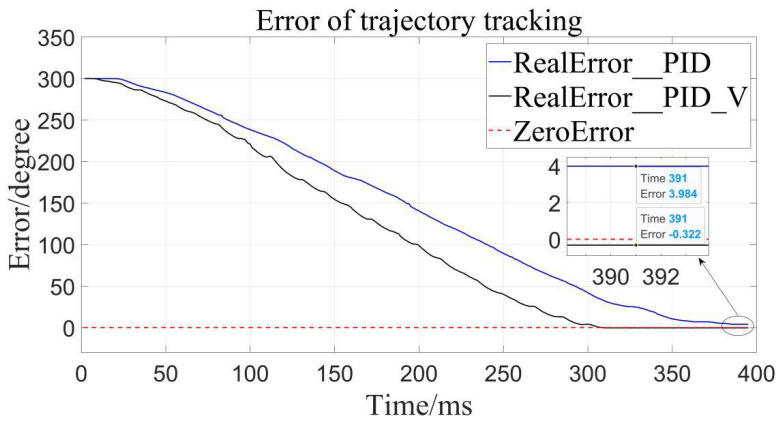
Error to target angle to 300°.

The results are as follows (Figure 8, Figure 9 and Figure 10), the caveat is that the blue solid line named RealAngle_PID represents the traditional PID control, the black solid line named RealAngle_PID_V represents the velocity-sensitive PID control, and the dotted line named TargetAngle represents the position-set point, the same below:

It can be clearly seen that the trajectory tracking (i.e., position loading) of the velocity-sensitive PID control is superior to the traditional PID control. In order to further quantify the gap, error analysis of the two is conducted (Figure 11, Figure 12 and Figure 13):

By calculation, the average error of traditional PID control is about 4.56°, and the relative error is 1.267% in the range of 0–360°, while the average error of the velocity-sensitive PID control is 0.231°, and its relative error is 0.064%. Using Table 2 given, it can be seen that precision of the latter is significantly higher than that of the former. Thus, position loading with velocity-sensitive control is feasible and has the distinct advantage of precision over previous methods.

Additionally, it can be clearly seen from the above trajectory tracking diagram that the setting time to steady state of the velocity-sensitive control is shorter than that of the traditional PID control. In other words, the improved PID control based on velocity-sensitive has a better rapidity than the traditional PID control for position loading.

The possible causes of the above situation are analyzed as follows:

Firstly, the static friction of the loading motor should be overcome at the beginning of rotation. That is, there is a torque of resistance during startup. As a result, in traditional PID control, when the input signal is in a small range near the zero value, the loading motor does not have the corresponding output signal, that is, there is a frictional dead zone which will cause uneven low-speed rotation of the system, and then lead to the imprecise trajectory tracking of the position servo system.

Secondly, there are backlashes between different rotary shafts of the loading device in the transmission mechanism, resulting in the nonlinear relationship between input and output in traditional PID control. The backlash characteristics will make the static error larger and affect the tracking precision of the position servo system.

By contrast, the velocity-sensitive control system does not have the influence of dead zone and backlash characteristics because it takes velocity as the reference value instead of displacement. The nonlinear system existing in the traditional PID control is transformed into a linear system in terms of variable velocity control, which improves the tracking precision of position servo system.

### 5.3. Torque Loading Experiment

The test method for torque loading is adding a magnetic powder clutch that can load torque within 25 N·m to the loading end of the test bench, as shown in Figure 7, so that the output shaft of the test bench is obstructed by torque under the condition of rotation or non-rotation, then driving the motor and controlling feedback of the transducers to load.

The torque loading performance of the designed adaptive robust controller is verified from two aspects of constant loading and load addition and subtraction, obtaining a constant loading figure and load addition and subtraction figure.

**Figure 14:**
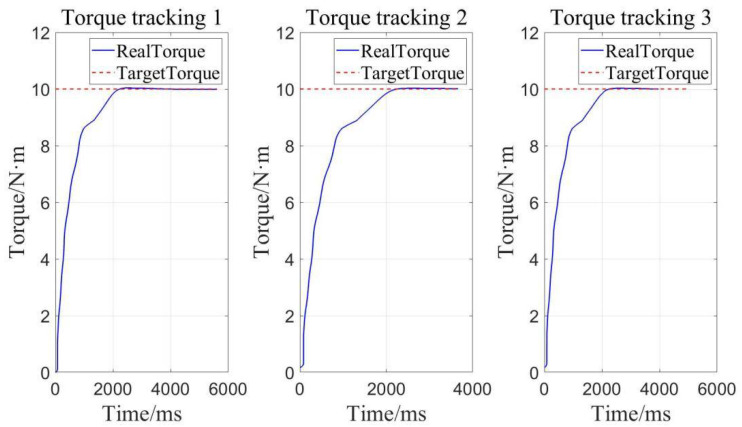
Determinate loading for torque tracking.

As shown in Figure 14 and Table 3, the average error of three constant torque loads is 0.003 N·m, and the relative error is 0.013% within the range of 0–25 N·m, when setting the constant loading target to 10 N·m. Obviously, it can be seen that the constant loading precision is close to 100% due to the adaptive robust controller.

**Figure 15:**
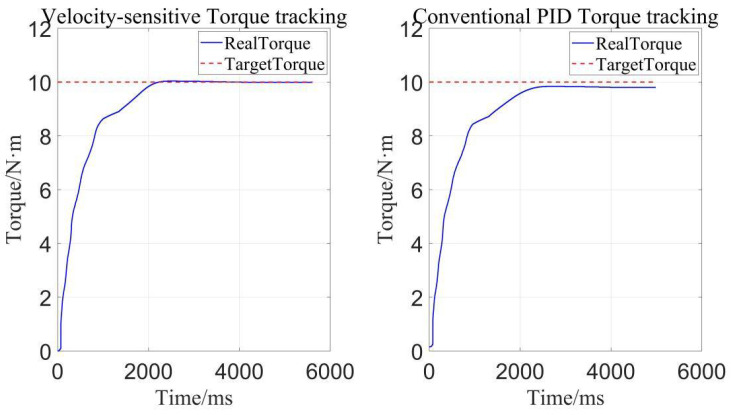
Influence of different PID controllers on the constant torque loading.

Here, we also explored the influence of improved PID control based on velocity sensitivity on the precision of constant torque loading. As we can see, in Figure 15, the improved PID control based on velocity sensitivity has more precise torque tracking than traditional PID control. That is to say, on the premise that adaptive robust controller plays a major role in torque tracking, improved PID control based on velocity-sensitive makes torque tracking more precise than before. Thus, improved PID control also exerts a positive impact on torque loading and it is indispensable in precise torque tracking.

**Figure 16:**
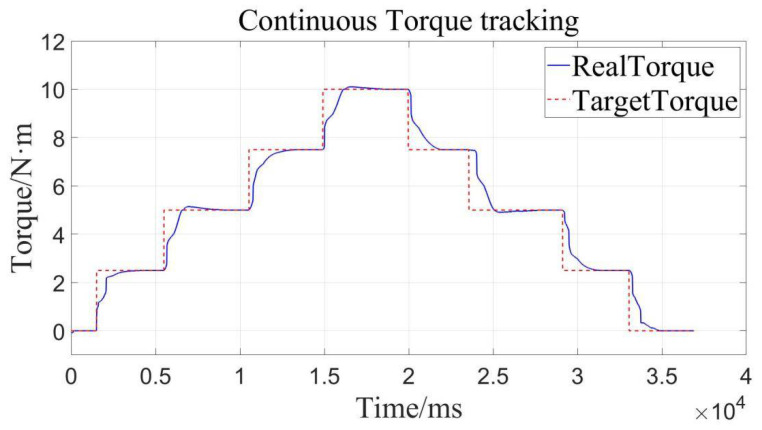
Load adding and shedding for torque tracking.

Load loading and unloading are carried out on the device with continuous torques of 0, 2.5, 5, 7.5, 10, 7.5, 5, 2.5, 0 (N·m), respectively. As can be seen from Figure 16 and Table 4, the device can precisely track the given target value well through loading and unloading, meaning that the precision is high in the loading and unloading process.

Here, we describe the zero errors of the load adding and shedding for torque tracking. From this, we can learn that the load adding and shedding system inherits the high-quality characteristics of the constant loading system, whose main reason is the combined action of the velocity-sensitive PID controller and the adaptive robust controller. That is to say, the precision, stability and rapidity of both controllers are all highlighted in the loading performance of the system, to a certain extent.

Through the constant loading and load adding and shedding tests above, the correctness of the theory in Section 4 is proved, and advantages of the torque loading system based on adaptive robust control are shown.

Furthermore, although there are practical and effective discoveries revealed by the research, our results suggest a possibility of more stable improvement due to the each section of continuous loading process is not exactly consistent, smooth and steady, which may be affected by the angle transducer of the velocity-sensitive controller and the torque transducer of the adaptive robust controller. Additionally, in the actual operation of the experiment, we also found that the experimental device did not run as smoothly as expected.

Thus, considering the complexity compared to conventional PID control, it is clear that the velocity-sensitive PID control is affected more than conventional PID control. Additionally, the potential problems from multiple groups of sensors working simultaneously are our next research direction. Additionally, the possible solutions such as sensor fusion, Kalman filtering, etc., maybe achieve more efficient stability than before. Thus, one significant future direction of the position and torque loading system with velocity-sensitive and adaptive robust control is to highly improve the working stability.

## 6. Conclusions

In this paper, the research of position and torque loading system based on velocity-sensitive and adaptive robust control is introduced. On the premise of considering factors that may affect its control performance, mathematical modeling and a corresponding controller are established to make the position and torque loading system precisely track the given trajectory and achieve precise loading.

In the position loading system, due to the influence of nonlinear factors such as dead zone characteristics, the velocity-sensitive controller proposed has better control performance than the traditional PID control for position loading. In addition, in the torque loading system, the controller shows good performance in both constant loading and load adding and shedding experiments, which further verifies the feasibility and advantages of the adaptive robust controller proposed.

In a word, the position and torque loading system with velocity-sensitive and adaptive robust control has certain advancement, where we demonstrate a significant improvement of tracking precision compared with traditional methods. The rapidity and precision of the velocity-sensitive controller has been well improved compared to a conventional PID controller. The simplicity of the design procedure and the low computation load makes the velocity-sensitive and adaptive robust control a potential off-the-shelf control technique for other loading systems.

## Figures and Tables

**Table 1:** Hardware of experiment platform.

Component	Model and Parameter
Loading motor	HT04/48V
Torque transducer	DYN-207/20Nm
Angular transducer	SE58T20-12-2-TACB-TG
Master computer	Thinkpad x1 Carbon
Slave computer	STM32F103
Power supply	48V
Magnetic powder clutch	0–25N·m

**Table 2:** Comparison of position errors of different controllers.

Control Method	Target Angel/Deg	Absolute Error/Deg	Average Error/Deg	Relative Error
Traditional PID control	100	8.330	4.560	1.267%
	200	1.367		
	300	3.984		
PID control based on Velocity-sensitive	100	0.244	0.231	0.064%
	200	0.127		
	300	0.322		

**Table 3:** Errors of target torque to 10N·m.

Target Torque /N·m	Real Torque /N·m	Absolute Error /N·m	Average Error /N·m	Relative Error
10	10	0	0.003	
	10.01	0.01		0.013%
	10	0		

**Table 4:** Errors of load adding and shedding for torque tracking.

Target Torque /N·m	Real Torque /N·m	Absolute Error /N·m
0	0	0
2.5	2.51	0.01
5	5.01	0.01
7.5	7.5	0
10	10	0
7.5	7.51	0.01
5	5	0
2.5	2.49	0.01
0	0	0

## Data Availability

Not applicable.
